# Home range, habitat use and capture-release of translocated leopards in Gir landscape, Gujarat, India

**DOI:** 10.1371/journal.pone.0305278

**Published:** 2024-06-10

**Authors:** Mohan Ram, Aradhana Sahu, Nityanand Srivastava, Rohit Chaudhary, Lahar Jhala, Yashpal Zala, Meena Venkataraman

**Affiliations:** 1 Wildlife Division, Sasan-Gir, Junagadh, Gujarat, India; 2 Wildlife Circle, Junagadh, Gujarat, India; 3 Chief Wildlife Warden, Gandhinagar, Gujarat, India; 4 Department of Wildlife Sciences, Navsari Agricultural University, Navsari, Gujarat, India; 5 Principal Consultant, Carnivore Conservation and Research, Mumbai, India; MARE – Marine and Environmental Sciences Centre, PORTUGAL

## Abstract

Understanding the spatial ecology of translocated leopards (*Panthera pardus fusca*) is crucial for their conservation and the effective assessment of conflict management strategies. We investigated the home range and habitat preferences of five radio-collared leopards (n = 5; 2 males; 3 females) in the Gir landscape. Additionally, we examined the usefulness of the capture-release strategy for these animals. We assessed home range and habitat selection using kernel density estimation (at 95% and 50% levels) and compositional analysis. Our findings revealed that leopards exhibited distinct patterns of movement, often returning to their original capture site or nearby locations or exploring new areas within 3 to 25 days, covering distances ranging from 48 to 260 km. The average home range (95% FK) was estimated at 103.96±36.37 (SE) km^2^, with a core area usage (50% FK) of 21.38±5.95 km^2^. Seasonally, we observed the largest home ranges during summer and the smallest during winter. Males exhibited larger home ranges (95% FK, 151±64.28 km^2^) compared to females (56.18±14.22 km^2^). The habitat analysis indicated that agricultural areas were consistently preferred in the multi-use landscape at the 2^nd^ order habitat selection level. Additionally, habitat around water bodies was highly favoured at the 3^rd^ order, with distinct variations in habitat selection observed during day and night. This study highlights the significance of riverine and scrubland habitats, as leopards exhibited strong preferences for these habitats within their home ranges. We emphasize the importance of conserving natural habitat patches, particularly those surrounding water bodies. We also report on the characteristics of the capture-release strategy and provide our observations indicating no escalated aggression by leopards’ post-release. In conclusion, this study evaluates widely employed approaches to conflict mitigation and suggests the continuous review and assessment of management strategies for mitigating human-leopard conflicts.

## Introduction

Conserving large carnivores in multi-use landscapes presents a significant challenge due to habitat loss and fragmentation. This is primarily because these landscapes often cannot meet the extensive spatial requirements necessary for the survival of large carnivores [1]. Although protected areas constitute a pivotal conservation strategy, they frequently fall short in meeting the spatial needs of these creatures due to limited size and slower establishment rates compared to habitat degradation [2]. Successful conservation efforts within protected areas may lead to an increase in large carnivore densities, resulting in their spillover into the surrounding landscapes [3]. These factors often result in the frequent utilization of multi-use landscapes adjoining protected areas by large carnivores.

Furthermore, the persistence of large carnivores in multi-use landscapes may pose threats to human populations and domestic livestock, thereby engendering negative perceptions among local communities [4]. Therefore, conserving large carnivores in multi-use landscapes necessitates robust scientific information to formulate effective conservation strategies [5].

Space constitutes a critical ecological resource essential for the survival of large carnivores. Large carnivores compete with humans for space in multi-use landscapes characterized by limited natural habitat availability [6]. Therefore, understanding the space utilization patterns of large carnivores in such landscapes is pivotal for their conservation and for devising strategies to mitigate human-large carnivore conflicts, a key concern in their conservation efforts [4].

Evaluating home ranges and habitat selection plays a crucial role in understanding the space used by large carnivores. Home range refers to the area utilized by an individual for routine activities, while habitat selection represents an adaptive behavior shaped over evolutionary time to maximize animal fitness [7, 8]. Given the substantial anthropogenic pressure in multi-use landscapes, understanding the adequacy and placement of home ranges can reveal how large carnivores balance the costs and benefits of resource utilization in such environments [9]. Moreover, habitat selection unfolds as a hierarchical process that aids in understanding the resources facilitating the persistence of large carnivores in multi-use landscapes. This selection can span multiple spatial scales: 1^st^ order (species range), 2^nd^ order (home range scale), 3^rd^ order (within the home range), and 4th order (foraging or resting areas) [10]. Alterations in the distribution of suitable habitat patches within multi-use landscapes can impact habitat selection and landscape-level connectivity, thereby influencing the home range selection process [11]. The inclusion of low-quality habitat patches within the home range may heighten energy expenditure for critical resource acquisition and potentially jeopardize the survival of large carnivores [6]. Consequently, the assessment of space utilization and habitat selection by large carnivores in multi-use landscapes holds paramount importance for the long-term viability of their populations. Furthermore, habitat selection by large carnivores may exhibit temporal variations in multi-use landscapes. Recent studies on pumas (*Puma concolor*) have demonstrated variations in habitat selection between day and night hours and found that daytime habitat patches free from human disturbance can act as source habitats in the multi-use landscape [11, 12].

Among large carnivores, the leopard (*Panthera pardus*) stands out as one of the most widely distributed felids and is known to persist in multi-use landscapes [13]. The Indian leopard (*Panthera pardus fusca*) is a species significantly implicated in human-wildlife conflicts, particularly in India [14]. The capture and relocation of leopards from conflict zones to alternative sites is a crucial management practice aimed at mitigating conflicts. Nevertheless, the strategy of capturing and releasing has a limited success rate and is not deemed as a long-term sustainable solution. Therefore, leopards in multi-use landscapes require information regarding their spatial ecology and capture and release strategy to support the conservation and management of human-leopard conflicts. The Saurashtra region of Gujarat, India, represents a multi-use landscape encompassing protected areas, agricultural land, pastoral land, industrial installations, and human settlements. The Gir National Park and Wildlife Sanctuary, the largest protected area in the Saurashtra landscape, sustains a remarkably high density of Indian leopards (19.90 leopards per 100 km^2^), leading to their dispersal into the surrounding multi-use landscape [15]. However, much of the available information on leopards is derived from Gir, with a lack of data from the surrounding multi-use Gir landscape [15–18]. The lack of information on multi-use landscapes hampers the development of conservation strategies for leopards and managing human-leopard conflicts. Additionally, the capture and release of problematic leopards constitute one of the strategies employed by Gir management authorities to moderate human-leopard conflicts and increase the tolerance of local people towards leopards.

Hence, this study seeks to elucidate Indian leopard space utilization in the multi-use Gir landscape while also assessing the effectiveness of leopard capture and release as a strategy for managing human-leopard conflicts. The objectives include: a) evaluating the home ranges of translocated leopards, b) assessing habitat selection by translocated leopards, and c) appraising the current conflict management strategy involving the capture and release of problematic individuals. We predicted that leopards would exhibit a positive association with native habitats over anthropogenic habitats, despite the latter’s higher availability.

## Materials and methods

### Study area

**Fig 1:**
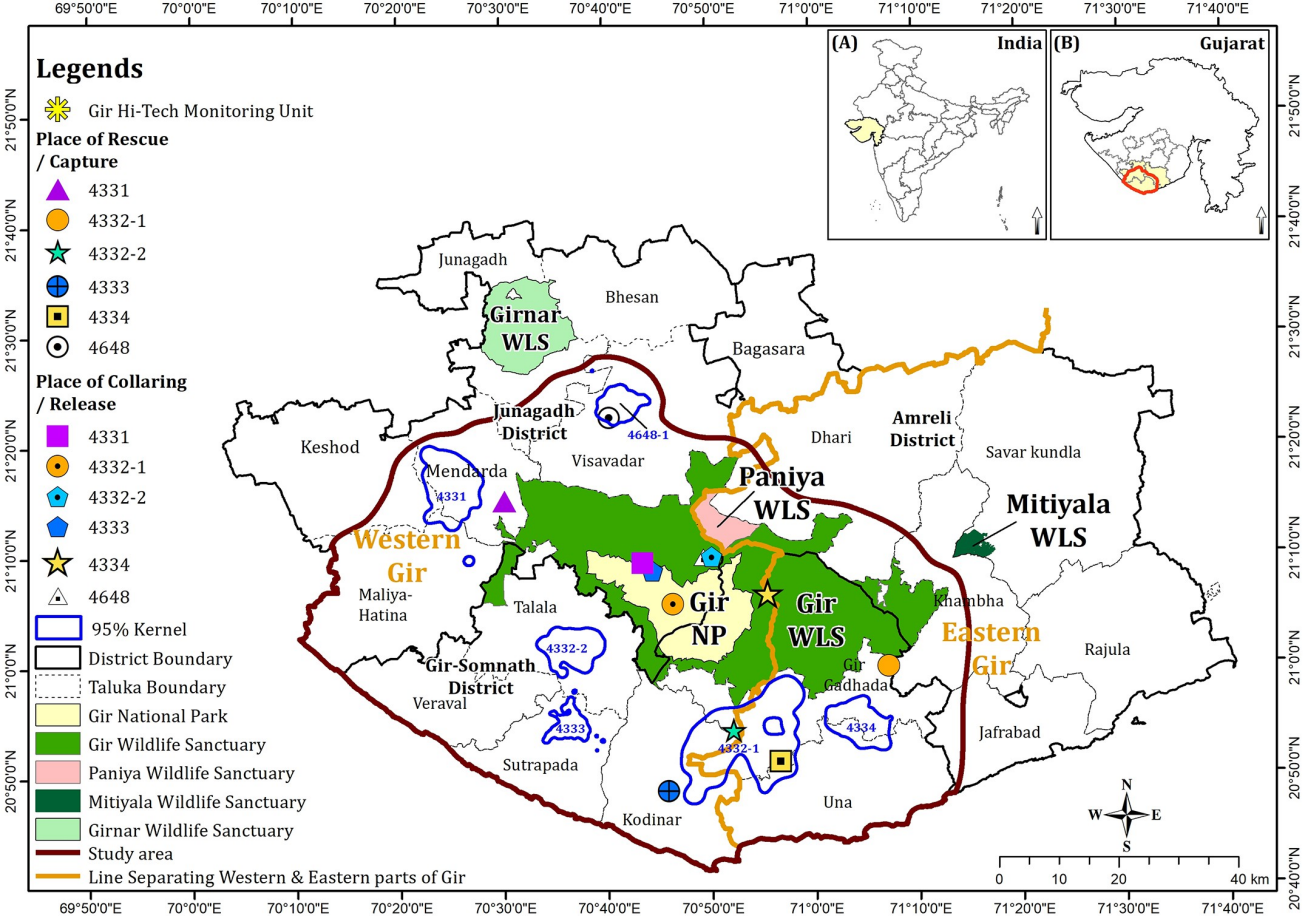
Map of the study area in Gir landscape, western India. The prominent star marks the Gir Hi-Tech Monitoring Unit at Sasan-Gir, a central unit for monitoring and data curation of radio-collared leopards. Numerical annotations in the legend specify radio-collar IDs and the capture/release locations for individual leopards. Inset maps provide additional context, showing Gujarat’s position in India (A) and the study area’s extent within Gujarat (B). We prepared the Fig 1 map by using a licenced version of ArcGIS 10.8.1 with base data from Bhaskaracharya National Institute for Space Applications and Geo-informatics (BISAG-N), Gandhinagar, Gujarat, officially sourced by Gujarat Forest Department, Government of Gujarat.

Gir, situated in the Saurashtra region of Gujarat state in western India, encompasses four protected areas: Gir National Park, Gir Wildlife Sanctuary, Paniya Wildlife Sanctuary, and Mitiyala Wildlife Sanctuary, as well as other peripheral forests such as reserved forests (RF), protected forests (PF), and unclassed forests (UF) [19]. The adjoining areas comprise revenue lands, fringe villages, croplands, wastelands, *Gaucher* (common grazing lands), and panchayat lands, collectively forming a multi-use land matrix surrounding Gir. For the purposes of this study, we will refer to this region as the Gir landscape (Fig 1).

The Gir landscape experiences three distinct seasons: summer (March-June), monsoon (July-October), and winter (November-February). It falls within a typical semi-arid biogeographical zone [20]. The primary source of precipitation in this region is the Indian southwest monsoon, contributing to an average annual rainfall of 976.50 mm (1996–2020) [19]. Natural ecosystems within the landscape encompass thorn-scrub forests, grasslands (known locally as *vidis*), dry deciduous forests, riverine forests, and coastal forests [21–24]. The Gir landscape predominantly supports agro-pastoral systems and boasts a diverse economy grounded in agriculture, horticulture, fisheries, animal husbandry, industry, and tourism.

Apart from leopards, the Gir landscape is also home to Asiatic lions (*Panthera leo persica*) and various other carnivorous species, including the striped hyena (*Hyaena hyaena*), Indian golden jackal (*Canis aureus*), jungle cat (*Felis chaus*), rusty-spotted cat (*Prionailurus rubiginosus*), and Indian fox (*Vulpes benghalensis*), among others [19]. The landscape’s most prevalent wild prey species comprise spotted deer (*Axis axis*), blue bulls (*Boselaphus tragocamelus*), sambar (*Rusa unicolor*), and wild pigs (*Sus scrofa*) [25].

### Methodology

#### Capture, selection, radio-collaring, release, and monitoring

Leopards were captured opportunistically based on requests/complaints from local villagers regarding conflict events on the forest fringes surrounding Gir (Fig 1). These selected individuals were trapped using baited cage traps (designed explicitly for leopard capture) at the conflict sites. Once captured, they were transported in specifically designed transport cages covered with green net via the shortest possible route to the nearest state-owned wildlife rescue centre.

For the study, adult males (n = 2) and females (n = 3) between the age of 5–10 years were chosen for radio-collaring. The captured individuals were immobilized using a combination of Ketamine (2.5–4.0 mg per kg body weight) and Xylazine (0.5–1.0 mg per kg body weight) administered intramuscularly. During sedation, each individual was sexed, aged, and measured following the Standard Operating Procedure (SOP) of the Gujarat Forest Department, Government of Gujarat (Table 1). Age estimation was based on tooth wear and other characteristics. All the individuals were deployed with lightweight GPS-GSM radio-collars (AWT GSM—Africa Wildlife Tracking), weighing less than 1.5% of the leopards’ body weight, regardless of age and sex. These collars had programmable GPS schedules that recorded location fixes every two hours. The data were downloaded from the AWT website (www.awt.co.za) and compiled at the Gir Hi-Tech Monitoring Unit, Sasan-Gir.

**Table 1:** Details of radio-collared leopards monitored from February 2021 to April 2022 in the Gir landscape.

Sr. No.	Particulars	Details of radio-collared Indian leopards					
		M1	M2-1 (Range I) *	M2-2 (Range II) *	F1	F2	F3
**1.**	**Sex**	Male	Male	Male	Female	Female	Female
**2.**	**Age (years)**	5–9	7–9	7–9	5–6	8–10	8–10
**3.**	**Range**^#^ **and division of capturing**	Dedakadi/Gir West	Tulshishyam/Gir East	Jamvala/Gir West	Jamvala/Gir West	Jasadhar/Gir East	Visavadar/Gir West
**4.**	**Date of radio-collaring**	22-02-2021	22-02-2021	11-12-2021	28-02-2021	24-03-2021	03-05-2021
**5.**	**Date of removal of collar/ Date till which the individual is monitored in this study**	30-04-2022^^^	09-11-2021	30-04-2022^^^	12-02-2022	30-04-2022^^^	22/09/2021
**6.**	**Date and place (range** ^#^ **) of returning to new/ original/ territory with a slight shift**	03-03-2021 (Dedakadi)^a^	26-02-2021 (Jamvala and Jasadhar)^b^	04-01-2022 (Talala)^b^	09-03-2021 (Veraval)^b^	02-04-2021 (Jasadhar)^b^	21-05-2021 (Visavadar)^c^
**7.**	**Distance moved from the place of release to new/ original/ territory with a slight shift (km)**	94.31	105.73	259.7	48.04	101.38	97.59
**8.**	**Total number of days taken to reach new/original/ territory with a slight shift from the place of release**	10	3	25	10	10	18
**9.**	**Number of villages within home range (100% MCP)**	35	53	16	32	26	15
**10.**	**Total number of monitoring days** ^$^	423	255	116	340	385	124
**11.**	**Overall (95% FK) home range (km** ^**2**^ **)**	103.4	244.90	64.16	30.88	66.46	38.74
**12.**	**Core (50% FK) home range (km** ^**2**^ **)**	24.5	44.6	13.08	3.75	14.55	9.76
**13.**	**Mean±SD (Range)–distance moved per day (km)**	8.7±4.36 (0.30–23.4)	6.2±4.4 (0.03–22.7)	8.2±4.4 (0.16–35.10)	3.8±2.4 (0.07–11.35)	4.0±2.8 (0.02–16.1)	4.22±2.6 (0.04–11.03)

M1, M2-1, M2-2 = adult males; F1, F2, F3 = adult females.

*Individual was captured a second time for injury treatment and released in the wild, establishing a new territory.

^#^Here, range means one of the administrative management units in the Gujarat Forest Department.

^$^On average, leopards were monitored for 273.8 days (±SD 131.7, range 116–423 days).

^a^-home range with a slight shift from the capture area;

^b^- home range away from captured locations;

^c^- home range established at captured locations.

^^^indicates that the individuals are still being monitored, and the collars are not removed.

After the deployment of radio-collars, the specific antidote Yohimbine (0.125 mg per kg body weight) was administered intravenously to facilitate the recovery of immobilized individuals within 5–10 minutes. Intensive monitoring was conducted for 72 hours post-collaring, followed by regular monitoring throughout the functional period of the collars. Qualified and experienced wildlife veterinary doctors and their teams conducted the radio-collaring procedure (Plate 1 to 5 in S1 File). All the permission required for radio-collaring was taken from the competent authority (Ministry of Environment, Forests & Climate Change, Government of India, New Delhi, letter No. F.No. 1-45/2019 WL dated 20 January 2020).

The collared individuals were released in Gir (Fig 1), and each one was assigned a unique code (Ref. ID) to avoid repetition and facilitate easy referencing in the paper. The Ref. IDs for the collared leopards are as follows: M1 for adult male-1 (collar # 4331), M2-1 for adult male-2 (collar # 4332–1) in Range I, M2-2 for adult male-2 (collar # 4332–2) in Range II, F1 for adult female-1 (collar # 4333), F2 for adult female-2 (collar # 4334), and F3 for adult female-3 (collar # 4648). It should be noted that M2, referred to as M2-1 and M2-2 leopards, established a new range after being captured for injury treatment, hospitalized for a month, and subsequently released. Therefore, M2 was captured and released twice, and the capture-release characteristics are summarized for both instances. Consequently, the leopard is referred to as M2-1 and M2-2, and the home range is reported as Range I and Range II (Table 1).

#### Habitat selection assessment

We used compositional analysis, as outlined by Aebischer et al. (1993) [26], to investigate the habitat utilization patterns by leopards across two spatial scales. This involved examining the utilization of land use categories within the leopard’s home range (defined as the 95% Minimum Convex Polygon) in comparison to the availability of such categories within the broader study area, a concept known as second-order selection (10). Additionally, we analysed the utilization of land cover categories at GPS location points in contrast to the available land use within the leopard’s home range, a concept referred to as third-order selection (10). To assess habitat selection at the second order, a super home range polygon was generated using the GPS locations of all radio-collared leopards. This super home range polygon represents the collective home range of all leopards, defined as the 100% minimum convex polygon and acts as availability of habitat for 2^nd^ order habitat selection [27]. Subsequently, this polygon was overlaid onto a spatial layer illustrating land use, thereby serving as a variable representing available habitats for the leopards at the home range scale.

The spatial layer depicting land use and land cover was categorized into six distinct habitat classes: agriculture, forest, scrubland, areas around water bodies (such as streams and rivers), orchards, and human settlements. By superimposing the super home range polygon onto this spatial layer, we quantified the proportion of each habitat class within the collective super home range of the leopards. This information facilitated an assessment of the availability of various habitat types and how they were utilized by leopards at the home range scale.

To evaluate habitat selection at the 3^rd^ order, we considered the land use classes within the home range of each leopard. The GPS locations of leopards in different habitat types within their respective home ranges were treated as the habitats utilized by those leopards. By comparing the utilized habitat (actual GPS locations of leopards) with the available habitat (habitat classes within their home range), we determined the habitat preferences and selection patterns exhibited by leopards at the third order.

### Data analysis

#### Movement and home range

In our study, individual leopard GPS data were processed and analyzed using ArcMap software (v. 10.8.1, ESRI, Redlands, USA). The GPS fixes were recorded at two-hour intervals, resulting in 12 daily fixes per leopard. We utilized the Tracking Analyst tool within ArcMap to calculate the average hourly distance (in meters) and the total distance covered in a day (in kilometers) by each leopard. These metrics provided valuable insights into the leopards’ movement characteristics, encompassing their journey from the release site to arrival at a stable territory. This entire episode, spanning from release to the moment of territorial settlement, was succinctly summarized in terms of the overall distance travelled and the number of days taken to reach the territory. Additionally, we computed the mean (±SE), maximum, and minimum daily distances moved per leopard. To ascertain if there were statistically significant differences in the mean daily distances travelled between male and female leopards, we conducted a two-sample t-test [28].

For estimating the home range of the leopards, we employed the fixed kernel (FK) method, implemented using the Adehabitat package within the R programming environment [29]. The FK method, initially proposed by Worton (1989) [30], is renowned for its robustness and unbiasedness as a home range estimator. It demonstrates excellent performance across varying sample sizes, utilization distribution shapes, autocorrelated data, and the presence of outliers. To determine the FK home range estimates, we utilized the least-square cross-validation (LSCV) method, known for its capacity to mitigate bias and yield well-distributed estimates [31]. In addition to calculating the standard 95% kernel estimate, which represents the typical home range, we also determined the 50% kernel estimate, indicating the core area extensively utilized by the leopards. To prevent temporal bias in our calculations, we considered two locations for every 24 hours, comprising one location from daytime and one from nighttime. This approach ensured that any unequal distribution of locations between day and night did not skew the home range estimates. We further used non-parametric Krukall-Wallis one-way ANOVA to test the statistical difference in seasonal home ranges of leopards [28].

#### Habitat selection analysis

To evaluate habitat selection by leopards at both the 2^nd^ and 3^rd^ orders, we conducted a compositional analysis using the Adehabitat package within the R programming [29]. Compositional analysis was chosen for two important reasons: firstly, it accounts for the non-independence of proportions, often referred to as the unit sum constraint, which can influence habitat selection assessments, and secondly, it addresses concerns related to inappropriate sampling levels and sample sizes [26].

The analysis involved comparing matrices of log-transformed use and availability distributions through a log-likelihood ratio test. This comparison determined whether the utilization of habitats by leopards was significantly non-random (p < 0.05). If significant non-random selection was identified, the habitat types were ranked based on the mean and standard deviation of log ratio differences for all habitat types, ordered from the most to the least selected [26].

At the 2^nd^ order, we assessed habitat availability within the collective super home range of all leopards. For the 3^rd^ order, we evaluated habitat availability within the individual home ranges of leopards. Furthermore, we categorized locations based on day and night hours to analyze habitat selection at the 3^rd^ order level with respect to diurnal and nocturnal periods. In certain instances, specific habitats within the study area had only a single leopard presence location at the 3^rd^ order level, posing a challenge for analysis. To address this concern, we followed the recommendation of Aebischer et al. (1993) [26] and replaced zero values in these particular habitats with a minimal value of 0.001.

#### Capture-release management

To understand the effectiveness of the capture-release management approach, our study involved not only closely tracking the movement of each leopard from the release site to its established resident territory but also closely monitoring the leopards’ behavior to detect any instances of aggressive interactions with human populations.

While our study is limited by a small sample size (n = 5), it is important to note that studying large carnivores often faces this challenge, as seen in peer-reviewed literature. Despite this constraint, the study’s outcome provides valuable insights into leopard spatial ecology and conflict management in the Gir landscape.

## Results

### Movement and home range

All radio-collared individuals released in Gir quickly ventured beyond the boundaries of Gir into the surrounding multi-use landscape. Of note, only one female leopard (F3) returned to its original capture site, and M1 displayed a slight shift from its initial range, while the others established new territories.

**Fig 2:**
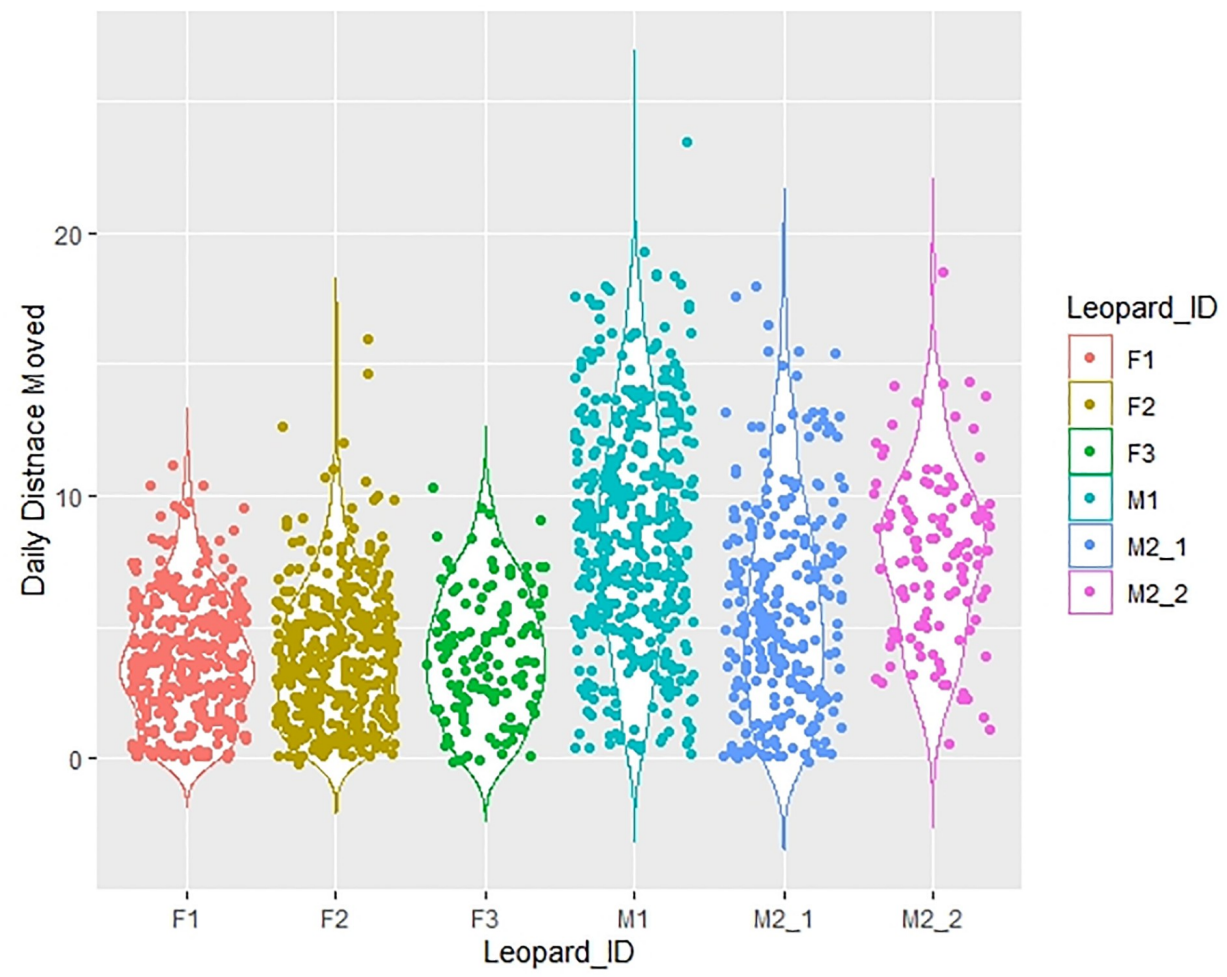
The summary of daily distance (km) moved by individual radio-collared leopards (M1, M2-1, M2-2, F1, F2, and F3). Notably, for leopard M2, the analysis considers two distinct ranges, denoted as Range I and Range II, each summarized as M2-1 (depicted in blue with dots) and M2-2 (depicted in pink with dots) movements, respectively.

The leopards displayed different lengths of travel time, ranging from 3 to 25 days, and travelled distances spanning from 48 to 260 km away from the release site (Table 1). Monitoring of male leopards continued for 787 days, while female leopards were monitored for 845 days. Among male leopards, M1 exhibited an average daily movement (km±SE) of 8.65±0.21 km, M2-1 moved 5.84±0.25 km, M2-2 covered 7.66±0.30 km, while for female leopards, F1 travelled 3.75±0.12 km, F2 moved 3.88±0.13 km, and F3 covered 4.03±0.21 km (as illustrated in Fig 2). The mean distance moved by males was 7.61±0.15 km, whereas, for females, it was 3.85±0.08 km (t = 1.96; p<0.05).

The average home range (95% FK) was found to be 103.96±36.37 (SE) km^2^ across both sexes. The core area usage (50% FK) amounted to 21.38±5.95 km^2^. Notably, male leopards exhibited larger average home ranges and core area usage when compared to their female counterparts (Table 2). Additionally, our observations revealed seasonal variations in leopard home ranges. Specifically, we noted that the average home range sizes (95% FK; 50% FK) for both sexes were at their highest during the summer season and at their lowest during the monsoon season, as outlined in Table 2. However, no significant difference was found in the 95% (H = 2.12, df = 4, p>0.05) and 50% (H = 2.86, df = 4, p>0.05) seasonal home ranges of leopards.

**Table 2:** Seasonal and pooled home range sizes of leopard males and females in Gir landscape, Gujarat, India.

Sex	95% FK±SE			50% FK±SE			Pooled±SE	
	Summer	Monsoon	Winter	Summer	Monsoon	Winter	95% FK	50% FK
**Male**	182.64 ± 102.34	125.97 ± 35.50	71.02 ± 21.14	51.80 ± 32.36	23.31 ± 1.42	18.20 ± 6.16	151 ± 64.28	30.59 ± 8.75
**Female**	77.22 ± 28.18	38.43 ± 13.78	53.72 ± 19.16	17.24 ± 5.85	8.76 ± 3.23	12.06 ± 6.10	56.18 ± 14.22	12.16 ± 3.97
**Pooled ± SE**	129.93 ± 53.00	61.20 ± 24.04	64.10 ± 13.74	34.62 ± 16.61	12.15 ± 4.07	13.12 ± 4.29	103.96 ± 36.37	21.38 ± 5.95

### Habitat selection

In the 2^nd^ order analysis, we found that leopard habitat selection differed significantly from the availability of habitats (λ = 0.10, p<0.05). The analysis indicated that the agriculture habitat was ranked the highest and was significantly selected more frequently than expected (p<0.05). Conversely, the forest habitat was ranked the lowest. It was significantly selected less frequently than expected, which could be attributed to its small size and human disturbances in forest patches in the multi-use landscapes (p<0.05) (Table 3).

**Table 3:** Habitat selection by leopards at 2^nd^ order scale in Gir landscape, Gujarat, India.

Habitat type	Habitat types and selection						
	Agriculture	Forest	Orchard	Scrub	Water Bodies	Human Settlements	^b^Rank
**Agriculture**	.	+++	+	+	+	+	5
**Human Settlements**	-	+++	+	+	+	.	4
**Scrub**	-	+	+	.	+	-	3
**Water Bodies**	-	+	+	-	.	-	2
**Orchard**	-	+++	.	-	-	-	1
**Forest**	—	.	—	-	-	—	0

Cells in the matrix consist of x¯ differences in the log ratios of used and available habitats for all leopards divided by the SE (i.e., t-values). The sign of the t-values is indicated with positive or negative signs, and triple signs represent a significant deviation from random at a ¼ 0.05;

^b^Rank is equal to the sum of the positive values in each row.

Higher ranks indicate a more preferred habitat.

In the 3^rd^ order analysis of habitat selection within individual leopard home ranges, as determined by their recorded locations, the compositional analysis unveiled a notable distinction in habitat selection during both daytime (λ = 1.6, p<0.05) and nighttime (λ = 8.57, p<0.05), as indicated in Table 4. During the daytime, water bodies were ranked the highest and were significantly (p<0.05) selected more frequently than expected, signifying a preference for these habitats. Conversely, human settlements were ranked the lowest and were significantly (p<0.05) selected less frequently than expected, indicating a lower preference for these areas during the day.

**Table 4:** Habitat selection at 3^rd^ order scale during day and night time in Gir landscape, Gujarat, India.

Habitat types	Habitat types and selection													
	Agriculture		Forest		Orchard		Scrub		Water Bodies		Human Settlements		^b^Rank	
	Day	Night	Day	Night	Day	Night	Day	Night	Day	Night	Day	Night	Day	Night
**Water Bodies**	+++	+++	+++	+++	+	+	+++	+	.	.	+++	+	5	5
**Scrub**	+	+++	+	+++	+	+	.	.	—	-	+++	+	4	4
**Orchard**	+++	+++	+++	+	.	.	-	-	-	-	+++	-	3	2
**Agriculture**	.	.	+	+	—	—	-	—	—	—	+	—	2	1
**Forest**	-	-	.	.	—	-	-	—	—	—	+	—	1	0
**Human Settlements**	-	+++	-	+++	—	+	—	-	—	-	.	.	0	3

Cells in the matrix consist of x¯ differences in the log ratios of used and available habitats for all leopards divided by the SE (i.e., t-values). The sign of the t-values is indicated with positive or negative signs, and triple signs represent a significant deviation from random at a ¼ 0.05;

^b^Rank is equal to the sum of the positive values in each row.

Higher ranks indicate a more preferred habitat.

During the nighttime, the habitat surrounding water bodies was ranked the highest and exhibited a significant preference, being selected more frequently than expected (p<0.05). Conversely, the forest habitat was ranked the lowest and was significantly selected less frequently than expected (p<0.05) during the night. Notably, the primary difference in habitat selection between daytime and nighttime was a decrease in the selection of agricultural habitats and an increase in the selection of human settlements during the nighttime hours.

### Radio-telemetry aided management

Our tracking and informed actions yielded significant benefits on several occasions during the study, helping the field staff and managers conserve leopards.

1. **Cub rescue**: When F1 gave birth to two cubs, we closely monitored the movement of the female and her habitat. Thanks to the alert from local people, we could rescue both cubs that had fallen into an open well. Radio-collar data and movement pattern analysis, promptly allowed us to confirm their identity as F1’s cubs and facilitate their swift reunion with their mother.
2. **Injury detection and wildlife veterinary care**: On another occasion, we detected the restricted movement of M2 due to an injury. This enabled us to rescue and provide wildlife veterinary care and treatment to the injured male leopard before his eventual re-release (as detailed in Table 2).
3. **Mortality verification**: Tragically, we verified the mortality of F3 through ground observations. Upon examination of circumstantial evidence, we determined that the individual had been electrocuted by a live fence on a private agricultural farm. A post-mortem conducted by a panel of wildlife veterinarians confirmed the cause of death as electrocution. Legal proceedings have been initiated against those responsible for this incident.

These instances illustrate how our monitoring efforts not only assist in law enforcement but also enhance our understanding of the various hazards wildlife species face outside of protected areas. This knowledge contributes to more effective conservation measures and wildlife protection.

## Discussion

### Home range and habitat selection

Understanding the spatial requirements and habitat preferences of leopards in multi-use landscapes is paramount for formulating effective conservation strategies. In this study, we observed notable variations in the average home range size (95% FK) of male and female leopards. Male leopards exhibited an average home range of 151±64.28 km^2^, which was comparatively smaller than the findings reported by Snider et al. (2021) [32] (male = 188.90±34.59 km^2^) in their comprehensive review of leopard space utilization within multi-use landscapes. Conversely, the average home range of female leopards in our study (56.18±14.22 km^2^) closely aligned with the estimates from Snider et al. (2021) [32] (58.26±10.52 km^2^). Notably, both male and female home ranges in our study exceeded those reported by Odden et al. (2014) [33] and Mondal et al. (2013) [34] in India. Additionally, Naha et al. (2021) [35] found similarly larger home ranges for leopards outside protected areas in India. The home ranges of leopards inside Gir National Park and Wildlife Sanctuary were far smaller (FK for males: 16–18 km^2^) than the present study [36].

Our findings correspond with those of Snider et al. (2021) [32], who observed that leopard home ranges in shared landscapes exhibit negative correlations with landscape productivity, human population density, and the interaction with habitat structure (open or closed). As our study area primarily featured with open semi-arid conditions, where agricultural crops such as cotton (*Gossypium* spp.), sugarcane (*Saccharum officinarum*), groundnut (*Arachis hypogaea*), sorghum (*Sorghum bicolor*), and millets were prevalent. However, only sugarcane offered dense ground cover, while the other crops had comparatively lower height and more open canopies. The necessity to locate suitable habitat patches in open cover conditions likely compelled leopards in our study to encompass larger home ranges compared to those in the study by Odden et al. (2014) [33].

Sexual dimorphism plays a pivotal role in home range disparities, with males typically exhibiting larger home ranges. This phenomenon is attributed to the larger body size and greater energetic demands of male leopards [37]. Additionally, the availability of mates and food resources significantly influences male home range size. For instance, McManus et al. (2021) [26] found that male leopards’ home range establishment in a South African multi-use landscape was influenced by proximity to female leopards’ home ranges.

Seasonal variations in home ranges were pronounced in our study, with larger home ranges observed during the summer compared to the monsoon and winter seasons. The expansion of summer home ranges can be attributed to reduced natural cover due to dry conditions, post-harvested crops, and lower cover in newly sowed crops. Moreover, for one reason or another, all the agricultural fields may not be ploughed for the summer crops, leaving some of these as fallow lands. Furthermore, water availability in the multi-use landscape likely contributed to larger home ranges during the summer. In contrast, the monsoon and winter seasons provided sufficient cover and water availability, reducing leopard home ranges.

Concerning habitat selection, our initial hypothesis was only partially supported in the 2^nd^ order analysis. Compositional analysis revealed that agricultural habitats and human settlements ranked first and second, respectively, while natural habitats such as forests ranked last. This result was not unexpected, considering that the individuals under study were opportunistically captured from the multi-use landscape and released into forest habitats in Gir. The homing-in instinct of large carnivores [3] and the high leopard density in Gir [15] likely influenced their movement within the surrounding multi-use landscape. Moreover, the limited availability of forest habitat in the multi-use landscape [19] contributed to forests ranking last.

Resource abundance, spatial distribution, and habitat quality influence large carnivores’ habitat selection at the home range scale [12]. In our study area, the high availability of agricultural land with dense crops like sugarcane, which provides suitable cover for leopards, likely contributed to their preference for agricultural habitats. Similar findings were reported by Naha et al. (2021) [35] in the multi-use landscape of North Bengal, India, where leopards used high tea gardens with dense understory cover. The multi-use Gir landscape featured natural habitats such as forests, scrubland, and habitat around water bodies in limited proportions, which were highly fragmented compared to agricultural land. Thus, maintaining home ranges exclusively comprising natural habitat was nearly impossible for leopards. This low availability of natural habitat likely contributed to its ranking last in 2^nd^ order habitat selection. We also noted female leopards using sugarcane fields to raise cubs, underscoring agricultural habitats’ crucial role in breeding. Following agricultural habitats, the high preference for human settlements in leopard home ranges, may be attributed to prey availability, including domestic dogs and small cats, which constitute a substantial prey base for leopards [38] in multi-use landscapes.

At the 3^rd^ order scale, we obtained partial support for our hypothesis, with habitat around water bodies ranking highest during both day and night. This finding aligned with McManus et al. (2021) [26], who also identified habitat around water bodies as a significant variable in leopard habitat selection. The dense habitat structure around water bodies offered refuge from human disturbances during the day and optimal hunting conditions for leopards [39]. Scrubland ranked high after habitat around water bodies during both day and night, likely due to the limited availability of riverine habitat. Nisi et al. (2023) [11] found a similar pattern when assessing the habitat selection of pumas. This suggests that daytime habitat selection around water bodies can serve as a proxy for source habitat in the multi-use Gir landscape. Orchards also emerged as important habitats for leopards during the daytime. Mango (*Mangifera indica*) orchards, prevalent in our study area, offer high canopy cover and potential daytime refuge for leopards, especially during non-peak cropping seasons. Furthermore, the first author’s observations indicate that mango orchards experience fewer disturbances, except for the peak cropping season from April to mid-June, compared to crops such as cotton. However, it is noteworthy that orchards did not exhibit the same pattern during nighttime hours. These findings provide additional support for the hypothesis that mango orchards serve as significant refuges during daylight hours.

Notably, habitat preferences shifted at night, with an increased use of human settlements compared to the day. This trend corresponds with findings in studies on pumas in Brazil [12]. High daytime disturbance levels may deter leopards from using human settlements, but at night, they may explore these areas for food and essential resources, including water. Interestingly, agricultural habitats ranked last during both day and night in 3^rd^ order habitat selection, despite their prominent role in 2^nd^ order selection. This suggests that leopards, while establishing their home ranges in extensive agricultural areas, still prefer less risky areas that offer refuge from human disturbance. Nonetheless, dense agricultural crops are essential for maintaining connectivity between natural habitat patches by facilitating movement.

### Capture-release management

The capture and translocation of large carnivores, such as leopards [34, 40, 41], black bears [42], and grey wolves [43], represent a widely employed strategy for mitigating human-wildlife conflicts. This approach serves dual purposes, ensuring the safety of the involved animals while also re-establishing a harmonious balance in the local ecosystem for the benefit of human communities [44]. Undoubtedly, translocation provides an immediate resolution at the site of a potential conflict incident. Yet, its significance extends beyond these individual cases, contributing to developing trust and collaboration between wildlife management authorities and local communities [44].

Assessing local attitudes towards Asiatic lions, leopards, and conservation revealed a notable disparity in favorability towards leopards. A majority of respondents expressed a preference for the removal of leopards from village areas [44]. Despite acknowledging the forest department’s efforts in addressing these conflicts through the ’capture-release’ strategy, the study indicated that this management technique had limited influence on local attitudes towards leopards in the Gir landscape [44].

Remarkably, our study revealed that all translocated leopards moved outside Gir, the release site. This finding can be attributed to the highly territorial nature of leopards, particularly in areas with high population densities. Gir boasts an exceptionally dense leopard population, with 19.90 individuals per 100 km^2^ [15], which may limit opportunities for new individuals to establish territories within the protected area. Moreover, the leopards exhibited a strong homing instinct, a behavior consistently observed in previous studies [39, 40]. This instinctual behavior, wherein leopards return to their original or nearby territories, is well-documented and further corroborated by our study’s findings, including observations of microchipped leopards.

Significantly, our monitoring efforts over a year during the study period did not yield reports of aggression or an escalation of negative interactions between these translocated leopards and humans. This suggests that the ’capture-release’ strategy did not exacerbate conflicts during the monitoring period, underlining its potential as an effective short-term mitigation option.

## Conclusion and conservation implications

Our study underscores important implications for leopard conservation in multi-use landscapes. The results emphasize the significance of natural habitats, particularly those around water bodies and scrublands, in leopards’ fine-scale resource selection. Adequate natural habitat patches within the agricultural expanse of the multi-use Gir landscape are imperative to effectively conserve leopards.

However, the intensification of agriculture and urban expansion pose threats to the persistence of natural habitats. Developing and implementing conservation strategies that address these challenges while ensuring the protection and conservation of riverine habitats and scrublands are vital for leopard survival. Special attention should be given to habitats around water bodies and scrublands, which serve as crucial daytime refuges and potential source habitats in multi-use landscapes.

The shift in cropping patterns surrounding Gir, particularly the transition from traditional crops to cash crops like sugarcane and mango, has proven advantageous for leopards by providing dense canopy cover and ground vegetation. Monitoring the influence of cropping patterns on leopard survival and habitat use is essential for understanding leopard population dynamics and conflict management in the multi-use Gir landscape.

Regarding leopard translocation, community attitudes toward leopards in the Gir landscape, particularly in comparison to endangered Asiatic lions, need to be factored into conflict management strategies, as these attitudes can influence the survival of Asiatic lions [43]. Given the ecological data and results, a more nuanced approach may be necessary for future "capture-release" efforts. Presently, in the absence of much-escalated conflicts, this approach may remain one of the most effective mitigation options available to managers.

Our study underscores the need for comprehensive and integrated conservation approaches that consider the intricate interactions between leopards, their habitats, and the surrounding multi-use landscapes. Addressing these challenges may enable the development of conservation strategies that ensure the long-term survival of leopards in such landscapes.

## Supporting information

S1 File Plates showing the process of radio-collaring, release and setup for remotely monitoring the radio-collared Indian leopards. All the images shared in Supplementary Information (S1_File) are the copyright of Wildlife Division, Sasan-Gir, Gujarat and are distrusted under CC BY 4.0 for free use with due credit.
(PDF)

S2 File Relevant data information used for the analysis in the paper titled "Home range, habitat use, and capture-release of translocated leopards in Gir landscape, Gujarat, India”. (PDF)
