# Supplementary material for: Home range, habitat use and capture-release of translocated leopards in Gir landscape, Gujarat, India
Source: PLoS One. 2024 Jun 10;19(6):e0305278. doi: 10.1371/journal.pone.0305278 (PMC11164372; doi:10.1371/journal.pone.0305278)
Supplement: S1 File — All the images shared in Supplementary Information (S1_File) are the copyright of Wildlife Division, Sasan-Gir, Gujarat and are distrusted under CC BY 4.0 for free use with due credit. (PDF) [file pone.0305278.s001.pdf]

Supporting Informing-1

Plates showing the process of radio-collaring, release and setup for remotely monitoring the radio-collared Indian leopards

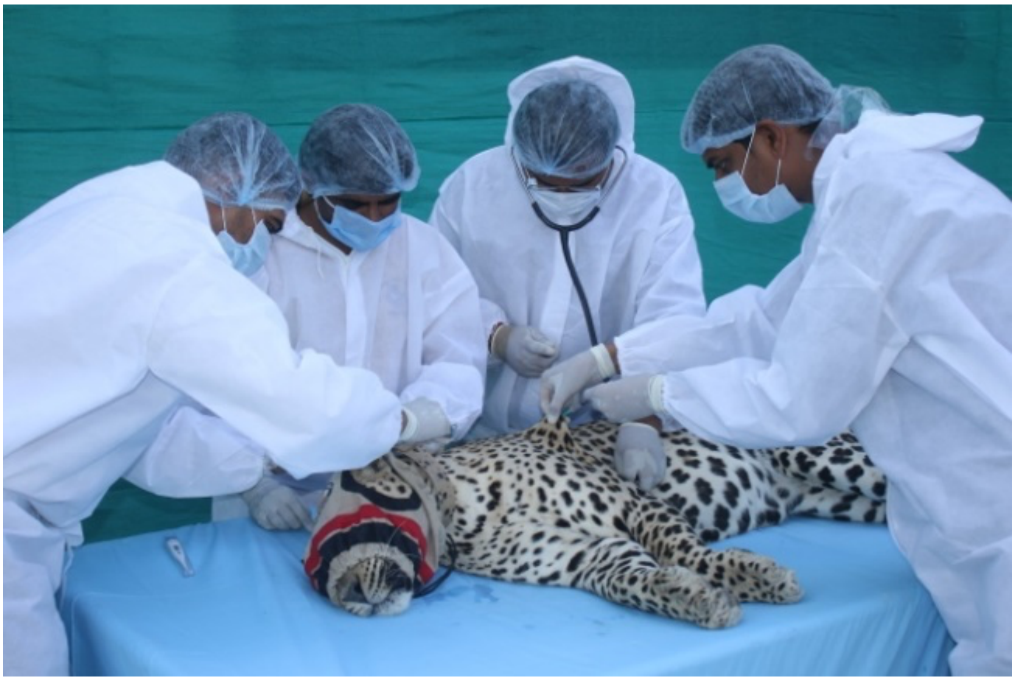

Plate 1: Team of experienced wildlife veterinarians deploying radio-collar on Indian leopard (*Panthera pardus fusca*).

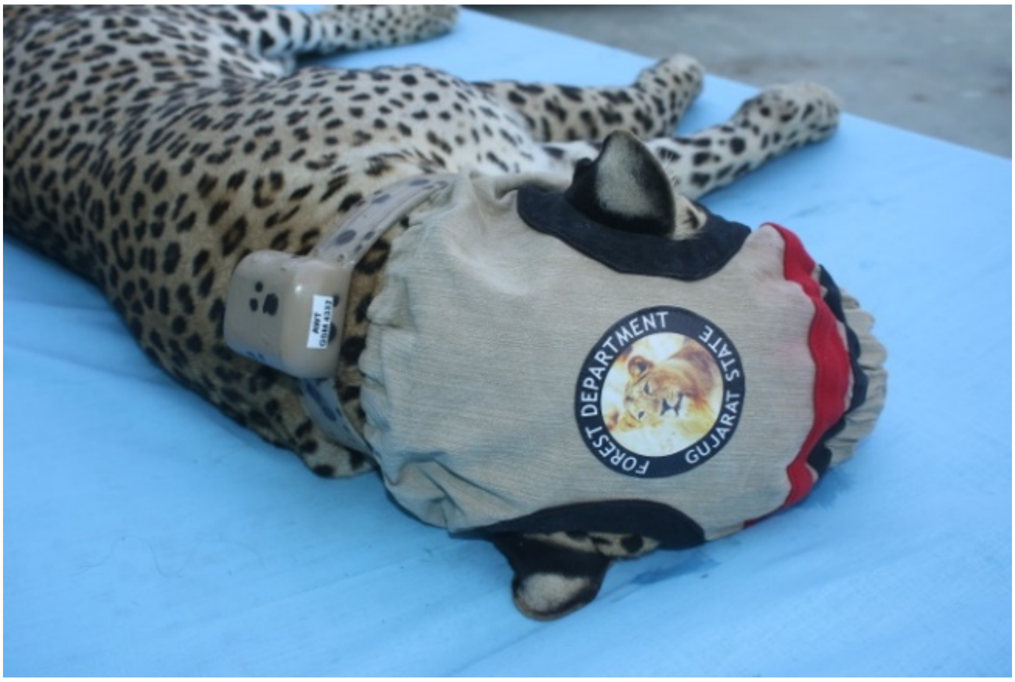

Plate 2: A male leopard after deployment of radio-collar.

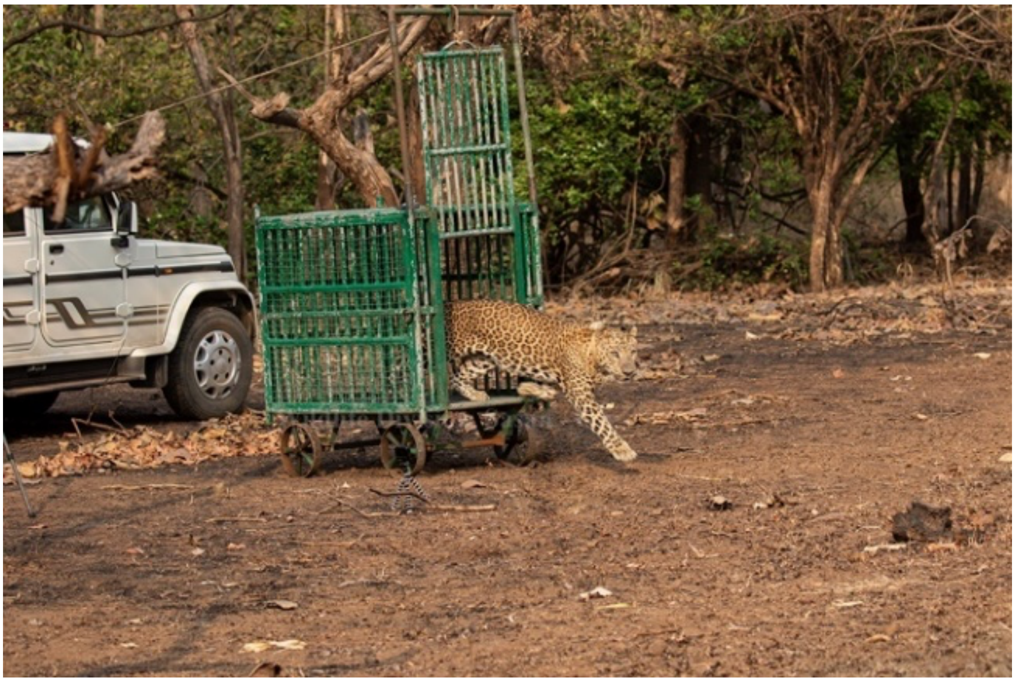

Plate 3: Leopard being released in the Gir forest.

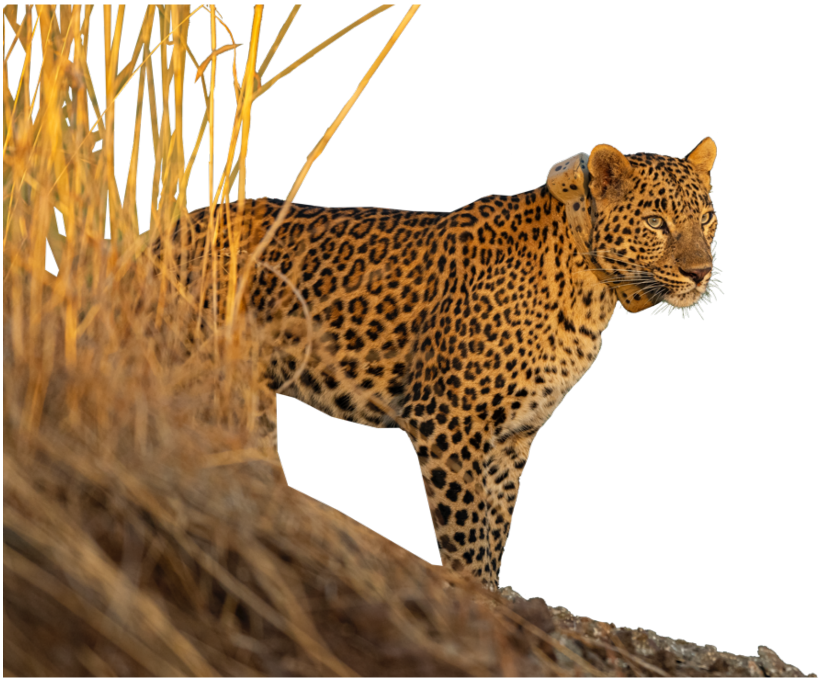

Plate 4: A radio-collared Indian leopard.

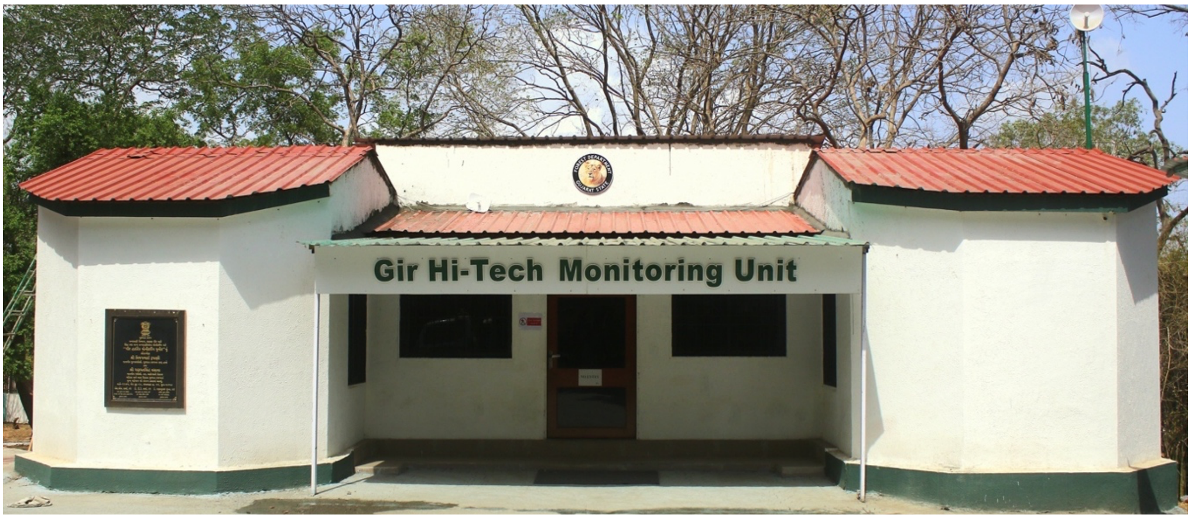

Plate 5: The setup of Gir Hi-Tech Monitoring Unit at Sasan-Gir, Gujarat, for data collection, analysis, and remotely monitoring the movement of radio-collared Indian leopards.

All the images shared in Supplementary Information (S1_File) are the copyright of Wildlife Division, Sasan-Gir, Gujarat and are distrusted under CC BY 4.0 for free use with due credit.

****
