# Supplementary material for: Home range, habitat use and capture-release of translocated leopards in Gir landscape, Gujarat, India
Source: PLoS One. 2024 Jun 10;19(6):e0305278. doi: 10.1371/journal.pone.0305278 (PMC11164372; doi:10.1371/journal.pone.0305278)
Supplement: S2 File — (PDF) [file pone.0305278.s002.pdf]

Supplementary Information-2

Relevant data information used for the analysis in the paper titled "Home range, habitat use, and capture-release of translocated leopards in Gir landscape, Gujarat, India.”

Supper Home Range Proportion			
**S.N.**	**LULC Class**	**Area (Sq. km)**	**Proportion**
1	Built Up	14.77	0.01
2	Cropland	1170.54	0.48
3	Forest	732.03	0.30
4	Horticulture	191.33	0.08
5	Settlements	50.98	0.02
6	Wasterlands	180.94	0.07
7	Waterbodies	74.3	0.03
		**2414.89**	

4331 Male (all location Use)			
**S.N.**	**Class**	**No. of location**	**Proportion**
1	Built Up	4	0.001
2	Cropland	1399	0.361
3	Forest	9	0.002
4	Horticulture	123	0.032
5	Settlement	97	0.025
6	Wastelands	1606	0.414
7	Waterbodies	637	0.164
		**3875**	

**4332-1 Male (all location Use)**			
**S.N.**	**Class**	**no of location**	**Proportion**
1	Built Up	37	0.015
2	Cropland	1559	0.627
3	Forest	58	0.023
4	Horticulture	95	0.038
5	Settlement	92	0.037
6	Wastelands	552	0.222
7	Waterbodies	92	0.037
		**2485**	

**4332-2 Male (all location Use)**			
**S.N.**	**Class**	**no of location**	**Proportion**
1	Built Up	17	0.015
2	Cropland	184	0.162
3	Forest	150	0.132
4	Horticulture	608	0.535
5	Settlement	7	0.006
6	Wastelands	87	0.077
7	Waterbodies	84	0.074
		**1137**	

**4333 Female (all location Use)**			
**S.N.**	**Class**	**no of location**	**Proportion**
1	Built Up	12	0.003
2	Cropland	2506	0.724
3	Forest	10	0.003
4	Horticulture	83	0.024
5	Settlement	76	0.022
6	Wastelands	617	0.178
7	Waterbodies	155	0.045
		**3459**	

4334 Female (all location Use)			
**S.N.**	**Class**	**no of location**	**Proportion**
1	Built Up	2	0.001
2	Cropland	2093	0.568
3	Forest	0	0.000
4	Horticulture	193	0.052
5	Settlement	145	0.039
6	Wastelands	830	0.225
7	Waterbodies	425	0.115
		**3688**	

4648 1 Female (all location Use)			
**S.N.**	**Class**	**no of location**	**Proportion**
1	Built Up	4	0.003
2	Cropland	566	0.469
3	Forest	0	0.000
4	Horticulture	17	0.014
5	Settlement	24	0.020
6	Wastelands	251	0.208
7	Waterbodies	344	0.285
		**1206**	

4331 Male (Night location Use)			
**S.N.**	**Class**	**no of location**	**Proportion**
1	Built Up	0	0.000
2	Cropland	99	0.434
3	Forest	3	0.013
4	Horticulture	11	0.048
5	Settlement	6	0.026
6	Wastelands	95	0.417
7	Waterbodies	14	0.061
		**228**	

4332-1 Male (Night location Use)			
S.N.	Class	no of location	Proportion
1	Built Up	1	0.005
2	Cropland	129	0.694
3	Forest	2	0.011
4	Horticulture	4	0.022
5	Settlement	9	0.048
6	Wastelands	35	0.188
7	Waterbodies	6	0.032
		**186**	

4332-2 Male (Night location Use)			
S.N.	Class	no of location	Proportion
1	Built Up	5	0.065
2	Cropland	18	0.234
3	Forest	11	0.143
4	Horticulture	26	0.338
5	Settlement	2	0.026
6	Wastelands	10	0.130
7	Waterbodies	5	0.065
		**77**	

4333 Female (Night location Use)			
S.N.	Class	no of location	Proportion
1	Built Up	1	0.004
2	Cropland	160	0.650
3	Forest	0	0.000
4	Horticulture	9	0.037
5	Settlement	13	0.053
6	Wastelands	53	0.215
7	Waterbodies	10	0.041
		**246**	

4334 Female (Night location Use)			
**S.N.**	**Class**	**no of location**	**Proportion**
1	Built Up	0	0.000
2	Cropland	170	0.669
3	Forest	0	0.000
4	Horticulture	5	0.020
5	Settlement	20	0.079
6	Wastelands	45	0.177
7	Waterbodies	14	0.055
		**254**	

4648 1 Female (Night location Use)			
**S.N.**	**Class**	**no of location**	**Proportion**
1	Built Up	0	0.000
2	Cropland	46	0.529
3	Forest	0	0.000
4	Horticulture	1	0.011
5	Settlement	9	0.103
6	Wastelands	13	0.149
7	Waterbodies	18	0.207
		**87**	

*****
